# Supplementary material for: The Equity and Effectiveness of Achieving Canada’s Voluntary Sodium Reduction Guidance Targets: A Modelling Study Using the 2015 Canadian Community Health Survey—Nutrition
Source: Nutrients. 2021 Feb 27;13(3):779. doi: 10.3390/nu13030779 (PMC7997239; doi:10.3390/nu13030779)
Supplement: Supplementary file 1 [file nutrients-13-00779-s001.pdf]

Online supporting material

Supplementary 1: Adjusting for dietary misreporting

To classify individuals according to level of dietary misreporting, respondents were categorized according to their ratio of total reported energy intake reported (EI) to total estimated energy requirements (EER)[1-3]. Children 12 years and under were categorized according to the following thresholds EI:EER <74% (under-reporters); 74% $\leq$ EI:EER $\leq$ 135% (plausible reporters); and EI:EER > 135% (over-reporters)[4]; respondents over 12 years were categorized based on the following established cut-points: EI:EER < 70% (under-reporters); 70% $\leq$ EI:EER $\leq$ 142% (plausible reporters), and EI:EER > 142% (over-reporters)[5,6]. EER was estimated using the Institute of Medicine factorial equations, including: age, sex, self-reported physical activity, height, and weight[1]. Following methods by Garriguet et al., when physical activity level was missing, the lowest physical activity level was assigned[7]. Correction equations for self-reported height and weight were applied to adult measurements[8]. If adults had no reported information on height and weight, we used the USDA equations to estimate daily EER. Respondents were grouped as "unclassified" if they could not be categorized due to missing information or reported as underweight.

References

1. National Academy Press, *Institute of Medicine. Dietary Reference Intakes for Energy, Carbohydrate, Fiber, Fat, Fatty Acids, Cholesterol, Protein and Amino Acids*. 2002: Washington DC.

2. Jessri, M., W.Y. Lou, and M.R. L'Abbe, *Evaluation of different methods to handle misreporting in obesity research: evidence from the Canadian national nutrition survey.* Br J Nutr, 2016. **115**(1): p. 147-59.

3. McCrory MA, H.C., Roberts SB, *Procedures for screening out inaccurate reports of dietary energy intake.* Public Health Nutrition, 2002. **5**: p. 873-882.

4Jessri, M., S.K. Nishi, and M.R. L'Abbe, *Assessing the nutritional quality of diets of Canadian children and adolescents using the 2014 Health Canada Surveillance Tool Tier System.* BMC Public Health, 2016. **16**: p. 381.

5. Hack, S., M. Jessri, and M.R. L'Abbe, *Evaluating Diet Quality of Canadian Adults Using Health Canada's Surveillance Tool Tier System: Findings from the 2015 Canadian Community Health Survey-Nutrition.* Nutrients, 2020. **12**(4).

6. Jessri, M., S.K. Nishi, and M.R. L'Abbe, *Assessing the Nutritional Quality of Diets of Canadian Adults Using the 2014 Health Canada Surveillance Tool Tier System.* Nutrients, 2015. **7**(12): p. 10447-68.

7. Garriguet, D., *Accounting for misreporting when comparing energy intake across time in Canada.* Health Reports, 2018. **29**(5): p. 3-12.

8. Shields, M., et al., *Bias in self-reported estimates of obesity in Canadian health surveys: an update on correction equations for adults.* Health Rep, 2011. **22**(3): p. 35-45.

Online Supporting Material

Supplementary Table S1. Adjusted mean dietary sodium intake (mg/day) according to DRI age-sex categories in the 2015 Canadian Community Health Survey-Nutrition (n=19,645).

	2015 CCHS-N Reported intake	Modelled intake		
	Measured (95% CI)	Phase 1 (95% CI)	Phase 2 (95% CI)	Phase 3 (95% CI)
**Children**				
2-3 years	1,729 (1,620, 1,837)	1,934 (1,839, 2,029)	1,823 (1,734, 1,912)	1,705 (1,622, 1,787)
4 - 8 years	2,297 (2,224, 2,370)	2,365 (2,294, 2,436)	2,214 (2,146, 2,281)	2,054 (1,991, 2,118)
**Male**				
9 - 13 years	2,959 (2,867, 3,052)	2,996 (2,902, 3,091)	2,801 (2,710, 2,892)	2,594 (2,507, 2,680)
14 - 18 years	3,323 (3,096, 3,551)	3,335 (3,048, 3,621)	3,127 (2,856, 3,399)	2,902 (2,650, 3,154)
19 - 30 years	3,405 (3,171, 3,639)	3,500 (3,256, 3,744)	3,277 (3,049, 3,504)	3,040 (2,828, 3,252)
31 - 50 years	3,424 (3,277, 3,571)	3,475 (3,316, 3,633)	3,264 (3,109, 3,419)	3,044 (2,893, 3,195)
51 - 70 years	2,896 (2,762, 3,030)	3,032 (2,906, 3,159)	2,838 (2,718, 2,958)	2,635 (2,520, 2,750)
> 70 years	2,585 (2,437, 2,734)	2,661 (2,499, 2,823)	2,494 (2,340, 2,649)	2,326 (2,178, 2,475)
**Female**				
9 - 13 years	2,579 (2,505, 2,652)	2,680 (2,594, 2,767)	2,509 (2,428, 2,589)	2,328 (2,254, 2,402)
14 - 18 years	2,460 (2,280, 2,640)	2,623 (2,439, 2,807)	2,466 (2,292, 2,640)	2,299 (2,134, 2,463)
19 - 30 years	2,315 (2,023, 2,608)	2,483 (2,235, 2,730)	2,330 (2,091, 2,569)	2,171 (1,941, 2,401)
31 - 50 years	2,593 (2,459, 2,728)	2,669 (2,533, 2,806)	2,502 (2,375, 2,628)	2,328 (2,212, 2,444)
51 - 70 years	2,216 (2,137, 2,296)	2,293 (2,213, 2,374)	2,150 (2,076, 2,225)	2,000 (1,931, 2069)
> 70 years	2,167 (2,067, 2,266)	2,210 (2,108, 2,312)	2,076 (1,978, 2,174)	1,935 (1,839, 2,030)

Results are weighted and bootstrapped according to Statistics Canada requirements using single 24-hour dietary recalls. Results are adjusted for continuous age, sex (children 2-8 years), total daily energy intake (kcal/day), and misreporting. “CCHS-N 2015” represents reported intake in the 2015 CCHS-N. “Phase 3” represents the effect of achieving Health Canada’s Voluntary Sodium Reduction Guidance.

Supplementary Table S2. Mean (95%CI) sodium intake (mg/day) by socioeconomic position in children 2 to 8 years from the 2015 Canadian Community Health Survey-Nutrition (n=2,171).

	2015 CCHS-N Reported intake		Modelled intake					
	Measured		Phase 1		Phase 2		Phase 3	
	Mean (95%CI)	Difference (95%CI)	Mean (95%CI)	Difference (95%CI)	Mean (95%CI)	Difference (95%CI)	Mean (95%CI)	Difference (95%CI)
**Food Security**								
Food insecure	2,335 (2,140, 2,531)	271 (75, 468)	2,296 (2,149, 2,444)	130 (-20, 280)	2,140 (2,004, 2,276)	106 (-32, 245)	1,976 (1,851, 2,102)	83 (-45, 212)
Food secure	2,064 (2,004, 2,124)	Ref.	2,166 (2,103, 2,229)	Ref.	2,033 (1,974, 2,093)	Ref.	1,893 (1,837, 1,949)	Ref.
**Household Education**								
Less than high school	2,379 (1,822, 2,936)	358 (-201, 916)	2,211 (1,908, 2,515)	54 (-249, 358)	2,089 (1,800, 2,378)	63 (-227, 353)	1,959 (1,685, 2,233)	72 (-204, 347)
High school only	2,233 (2,034, 2,432)	212 (5, 419)	2,254 (2,127, 2,382)	97 (-35, 229)	2,106 (1,989, 2,222)	80 (-40, 200)	1,949 (1,843, 2,055)	61 (-48, 171)
Trade, college etc.	2,126 (2,045, 2,208)	105 (9, 201)	2,185 (2,088, 2,282)	28 (-74, 130)	2,048 (1,957, 2,139)	22 (-75, 118)	1,904 (1,818, 1,990)	16 (-74, 106)
Bachelor degree etc.	2,021 (1,948, 2,095)	Ref.	2,157 (2,094, 2,221)	Ref.	2,026 (1,966, 2,086)	Ref.	1,888 (1,831, 1,944)	Ref.
**Income**								
Quintile 1	2,151 (2,026, 2,276)	170 (-1, 342)	2,204 (2,102, 2,307)	75 (-74, 223)	2,064 (1,968, 2,159)	62 (-76, 199)	1,914 (1,826, 2,002)	49 (-77, 175)
Quintile 2	2,070 (1,949, 2,192)	90 (-68, 249)	2,225 (2,102, 2,347)	95 (-66, 255)	2,092 (1,976, 2,207)	90 (-59, 239)	1,952 (1,844, 2,061)	87 (-49, 224)
Quintile 3	2,132 (2,015, 2,249)	151 (3, 299)	2,193 (2,086, 2,301)	63 (-80, 207)	2,059 (1,958, 2,159)	57 (-76, 190)	1,917 (1,822, 2,011)	52 (-71, 174)
Quintile 4	2,060 (1,981, 2,140)	80 (-54, 214)	2,120 (2,044, 2,197)	-10 (-148, 128)	1,985 (1,913, 2,056)	-17 (-145, 111)	1,842 (1,774, 1,911)	-23 (-141, 96)
Quintile 5	1,980 (1,863, 2,098)	Ref.	2,130 (2,013, 2,247)	Ref.	2,002 (1,894, 2,109)	Ref.	1,865 (1,767, 1,963)	Ref.

Results are weighted and bootstrapped according to Statistics Canada requirements using single 24-hour dietary recalls. Results are adjusted for continuous age, sex, total daily energy intake (kcal/day), and misreporting. “Measured” represents reported intake in the 2015 CCHS-N.

Supplementary Table S3. Mean sodium intake (mg/day) by socioeconomic position in 9-18 years respondents from the 2015 Canadian Community Health Survey-Nutrition (n=3,955).

	2015 CCHS-N Reported intake		Modelled intake					
	Measured		Phase 1		Phase 2		Phase 3	
	Mean (95%CI)	Difference (95%CI)	Mean (95%CI)	Difference (95%CI)	Mean (95%CI)	Difference (95%CI)	Mean (95%CI)	Difference (95%CI)
**Males 9 -18 years**								
**Food Security**								
Food insecure	3,138 (2,937, 3,339)	-19 (-227, 189)	3,144 (2,959, 3,329)	-55 (-243, 143)	2,930 (2,756, 3,105)	-64 (-241, 112)	2,711 (2,547, 2,875)	-66 (-232, 100)
Food secure	3,157 (3,052, 3,263)	Ref.	3,198 (3,079, 3,318)	Ref.	2,995 (2,881, 3,108)	Ref.	2,777 (2,670, 2,883)	Ref.
**Household Education**								
Less than high school	3,249 (2,949, 3,550)	148 (-161, 456)	2,997 (2,685, 3,309)	-188 (-510, 135)	2,783 (2,484, 3,083)	-203 (-512, 105)	2,562 (2,272, 2,853)	-211 (-509, 87)
High school only	3,115 (2,939, 3,290)	13 (-175, 201)	3,201 (3,039, 3,363)	16 (-142, 175)	2,999 (2,847, 3,151)	13 (-136, 161)	2,785 (2,643, 2,926)	12 (-129, 152)
Trade, college etc.	3,223 (3,084, 3,362)	121 (-34, 277)	3,218 (3,076, 3,360)	33 (106, 172)	3,006 (2,872, 3,140)	20 (-111, 151)	2,780 (2,655, 2,905)	7 (118, 131)
Bachelor degree etc.	3,102 (2,974, 3,229)	Ref.	3,185 (3,047, 3,323)	Ref.	2,986 (2,856, 3,117)	Ref.	2,773 (2,651, 2,896)	Ref.
**Income**								
Quintile 1	3,052 (2,892, 3,211)	-224 (-442, -5)	3,126 (2,963, 3,289)	-65 (-273, 142)	2,926 (2,772, 3,081)	-65 (-263, 133)	2,718 (2,573, 2,863)	-56 (-244, 133)
Quintile 2	3,069 (2,923, 3,214)	-207 (-423, 10)	3,240 (3,076, 3,405)	49 (-170, 268)	3,033 (2,877, 3,189)	41 (-168, 250)	2,814 (2,666, 2,962)	41 (-159, 240)
Quintile 3	3,262 (3,105, 3,419)	-14 (-245, 217)	3,230 (3,085, 3,376)	39 (-166, 245)	3,025 (2,890, 3,160)	34 (-161, 228)	2,803 (2,679, 2,928)	30 (153, 213)
Quintile 4	3,118 (2,947, 3,290)	-157 (-391, 78)	3,169 (2,961, 3,377)	-22 (-249, 204)	2,957 (2,761, 3,152)	-35 (-250, 180)	2,731 (2,550, 2,912)	-43 (-245 160)
Quintile 5	3,275 (3,078 ,3,472)	Ref.	3,191 (3,000, 3,382)	Ref.	2,992 (2,809, 3,175)	Ref.	2,773 (2,600, 2,947)	Ref.
**Females 9 -18 years**								
**Food Security**								
Food insecure	2,636 (2,469, 2,803)	122 (-52, 296)	2,657 (2,498, 2,816)	19 (-142, 180)	2,479 (2,329, 2,629)	6 (-146, 158)	2,295 (2,153, 2,436)	-4 (-149, 140)
Food secure	2,514 (2,434, 2,593)	Ref.	2,639 (2,553, 2,724)	Ref.	2,473 (2,393, 2,553)	Ref.	2,299 (2,224, 2,374)	Ref.
**Household Education**								
Less than high school	2,740 (2,498, 2,981)	246 (-1, 493)	2,790 (2,526, 3,054)	113 (-156, 382)	2,621 (2,369, 2,873)	106 (-151, 363)	2,442 (2,202, 2,682)	96 (-149, 341)
High school only	2,523 (2,394, 2,652)	29 (-127, 185)	2,605 (2,478, 2,732)	-72 (-225, 81)	2,434 (2,315, 2,552)	-82 (-226, 63)	2,256 (2,146, 2,365)	-90 (-227, 46)
Trade, college etc.	2,535 (2,430, 2,641)	41 (-86, 169)	2,599 (2,488, 2,709)	-79 (-214, 57)	2,429 (2,327, 2,531)	-87 (-214, 41)	2,250 (2,156, 2,344)	-96 (-216, 24)
Bachelor degree etc.	2,494 (2,387, 2,601)	Ref.	2,677 (2,563, 2,791)	Ref.	2,515 (2,407, 2,624)	Ref.	2,346 (2,243, 2,449)	Ref.
**Income**								
Quintile 1	2,521 (2,378, 2,663)	15 (-172, 201)	2,705 (2,562, 2,847)	115 (-75, 306)	2,539 (2,404, 2,674)	109 (-70, 287)	2,366 (2,238, 2,495)	104 (-63, 271)
Quintile 2	2,617 (2,464, 2,771)	111 (-90, 312)	2,726 (2,566, 2,886)	136 (-82, 354)	2,555 (2,403, 2,706)	125 (-80, 329)	2,375 (2,232, 2,519)	113 (-79, 304)
Quintile 3	2,532 (2,403, 2,660)	26 (-143, 194)	2,619 (2,485, 2,753)	30 (-148, 207)	2,447 (2,323, 2,571)	17 (-149, 182)	2,267 (2,153, 2,382)	5 (-149, 159)
Quintile 4	2,421 (2,312, 2,530)	-85 (-249, 79)	2,519 (2,399, 2,638)	-71 (-255, 114)	2,357 (2,244, 2,470)	-73 (-246, 100)	2,185 (2,078, 2,292)	-78 (-238, 83)
Quintile 5	2,506 (2,370, 2,643)	Ref.	2,589 (2,434, 2,745)	Ref.	2,430 (2,286, 2,574)	Ref.	2,262 (2,129, 2,396)	Ref.

Results are weighted and bootstrapped according to Statistics Canada requirements using single 24-hour dietary recalls. Results are adjusted for continuous age, total daily energy intake (kcal/day), and misreporting. “Measured” represents reported intake in the 2015 CCHS-N.

Supplementary Table S4. Sex-specific mean sodium intake (mg/day) by socioeconomic position in respondents ≥ 19 years respondents from the 2015 Canadian Community Health Survey-Nutrition (n=13,519).

	2015 CCHS-N Reported intake		Modelled intake					
	Measured		Phase 1		Phase 2		Phase 3	
	Mean (95%CI)	Difference (95%CI)	Mean (95%CI)	Difference (95%CI)	Mean (95%CI)	Difference (95%CI)	Mean (95%CI)	Difference(95%CI)
**Males ≥ 19 years**								
**Food Security**								
Food insecure	3,137 (2,910, 3,365)	48 (-186, 282)	2,994 (2,799, 3,190)	-199 (-396, -2)	2,800 (2,616, 2,984)	-193 (-380, -7)	2,596 (2,423, 2,770)	-188 (-365, -11)
Food secure	3,089 (2,996, 3,182)	Ref.	3,193 (3,105, 3,282)	Ref.	2,993 (2,908, 3,077)	Ref.	2,784 (2,703, 2,865)	Ref.
**Education**								
Less than high school	3,400 (3,186, 3,614)	399 (172, 626)	3,332 (3,144, 3,520)	163 (-35, 361)	3,128 (2,950, 3,306)	155 (-33, 342)	2,918 (2,748, 3,087)	148 (-30, 327)
High school only	3,177 (3,045, 3,309)	176 (26, 325)	3,162 (3,035, 3,290)	-7 (-147, 133)	2,962 (2,843, 3,080)	-12 (-144, 120)	2,751 (2,641, 2,861)	-18 (-143, 107)
Trade, college etc.	3,076 (2,969, 3,184)	75 (-51, 201)	3,158 (3,055, 3,261)	-11 (-126, 103)	2,956 (2,859, 3,053)	-18 (-127, 92)	2,745 (2,654, 2,837)	-24 (-130, 82)
Bachelor degree etc.	3,001 (2,876, 3,126)	Ref.	3,169 (3,054, 3,285)	Ref.	2,973 (2,861, 3,085)	Ref.	2,769 (2,660, 2,878)	Ref.
**Income**								
Quintile 1	3,113 (2,933, 3,293)	82 (-126, 290)	3,224 (3,071, 3,378)	154 (-27, 335)	3,021 (2,872, 3,169)	138 (-35, 311)	2,811 (2,667, 2,954)	126 (-40, 292)
Quintile 2	3,083 (2,949, 3,217)	52 (-118, 222)	3,194 (3,066, 3,323)	124 (-38, 286)	2,991 (2,871, 3,112)	108 (-45, 262)	2,781 (2,668, 2,893)	96 (-49, 241)
Quintile 3	3,129 (2,999, 3,259)	98 (-56, 252)	3,233 (3,113, 3,352)	162 (14, 311)	3,027 (2,915, 3,139)	144 (5, 284)	2,814 (2,708, 2,919)	129 (-2, 260)
Quintile 4	3,120 (2,984, 3,256)	89 (-74, 251)	3,183 (3,047, 3,318)	112 (-46, 270)	2,981 (2,854, 3,107)	98 (-52, 247)	2,770 (2,651, 2,889)	86 (-56, 227)
Quintile 5	3,031 (2,887, 3,175)	Ref.	3,071 (2,930, 3,211)	Ref.	2,883 (2,748, 3,018)	Ref.	2,685 (2,555, 2,814)	Ref.
**Females ≥ 19 years**								
**Food Security**								
Food insecure	2,276 (2,168, 2,385)	-67 (-172, 38)	2,295 (2,191, 2,400)	-133 (-236, -31)	2,154 (2,055, 2,254)	-123 (-221, -25)	2,007 (1,912, 2,101)	-112 (-206, -18)
Food secure	2,343 (2,280, 2,406)	Ref.	2,428 (2,365, 2,492)	Ref.	2,277 (2,218, 2,337)	Ref.	2,119 (2,064, 2,175)	Ref.
**Education**								
Less than high school	2,412 (2,306, 2,517)	122 (6, 239)	2,368 (2,270, 2,466)	-52 (-164, 60)	2,220 (2,128, 2,311)	-49 (-153, 54)	2,067 (1,981, 2,152)	-45 (-141, 51)
High school only	2,358 (2,266, 2,451)	69 (-42,180)	2,421 (2,334, 2,508)	1 (-108, 111)	2,269 (2,187, 2,351)	0 (-102, 102)	2,110 (2,032, 2,188)	-2 (-97, 94)
Trade, college etc.	2,353 (2,273, 2,433)	64 (-38, 166)	2,426 (2,342, 2,509)	6 (-97, 109)	2,276 (2,198, 2,354)	7 (-89, 102)	2,119 (2,047, 2,191)	7 (-81, 94)
Bachelor degree etc.	2,289 (2,199, 2,380)	Ref.	2,420 (2,331, 2,509)	Ref.	2,269 (2,186, 2,352)	Ref.	2,112 (2,035, 2,188)	Ref.
**Income**								
Quintile 1	2,305 (2,206, 2,404)	-41 (-184, 103)	2,401 (2,304, 2,498)	-9 (-165, 146)	2,255 (2,165, 2,346)	4 (-140, 148)	2,105 (2,021, 2,189)	21 (-111, 154)
Quintile 2	2,313 (2,225, 2,401)	-33 (-171, 106)	2,427 (2,342, 2,513)	17 (-127,162)	2,271 (2,191, 2,352)	20 (-113, 153)	2,110 (2,034, 2,185)	26 (-95, 146)
Quintile 3	2,411 (2,312, 2,509)	65 (-82, 211)	2,463 (2,372, 2,554)	53 (-96, 201)	2,312 (2,226, 2,398)	61 (-77, 198)	2,153 (2,072, 2,234)	70 (-57, 195)
Quintile 4	2,321 (2,220, 2,423)	-25 (-182, 133)	2,381 (2,286, 2,477)	-29 (-192, 134)	2,240 (2,150, 2,330)	-11 (-162, 140)	2,092 (2,007, 2,177)	8 (-131, 147)
Quintile 5	2,346 (2,222, 2,470)	Ref.	2,410 (2,275, 2,546)	Ref.	2,251 (2,126, 2,377)	Ref.	2,084 (1,969, 2,199)	Ref.

Results are weighted and bootstrapped according to Statistics Canada requirements using single 24-hour dietary recalls. Results are adjusted for continuous age, total daily energy intake (kcal/day), and misreporting. “Measured” represents reported intake in the 2015 CCHS-N.

Supplementary Table S5. Percent of Canadians above the chronic disease risk reduction (CDRR) threshold for sodium intake by age, sex and socioeconomic position as measured and modelled full compliance to Health Canada's voluntary Sodium Reduction Guideline (SRG) targets for processed food using data from the 2015 Canadian Community Health Survey-Nutrition (n=19,645).

	**Children 2-8 years**		**Males 9-18 years**		**Females 9-18 years**		**Males ≥ 19 years**		**Females ≥ 19 years**	
	**Measured**	**Modelled**	**Measured**	**Modelled**	**Measured**	**Modelled**	**Measured**	**Modelled**	**Measured**	**Modelled**
	**Percent above the CDRR (95%CI)**									
**Total**	89%(83, 94)	79% (74, 84)	87% (82, 93)	76% (71, 82)	63% (57, 70)	48% (42, 54)	79% (76, 82)	71% (67, 74)	47% (44, 50)	34% (31, 37)
**Household Education**										
Less than high school	89% (82, 97)	80% (71, 89)	83% (73,92)	69% (57,80)	62% (53,70)	46% (37,54)	74% (69,78)	63% (58,68)	39% (34,43)	25% (22,29)
High school only	88% (82, 95)	79% (73, 85)	88% (82,93)	77% (70,83)	62% (56,69)	47% (41,54)	76% (73,80)	68% (64,72)	44% (40,47)	31% (28,35)
Trade, college etc.	89% (83, 95)	80% (74, 85)	86% (81,92)	75% (69,81)	64% (57,70)	48% (42,54)	80% (77,83)	72% (69,76)	48% (44,51)	35% (31,38)
Bachelor degree etc.	88% (82, 94)	79% (73, 84)	88% (83,94)	78% (72,83)	64% (57,71)	49% (42,55)	80% (76,83)	72% (68,76)	49% (45,52)	36% (32,39)
**Income**										
Quintile 1	89% (83, 95)	80% (74, 85)	87% (81,93)	76% (69,82)	62% (55,69)	47% (40,53)	80% (76,83)	72% (67,76)	43% (40,47)	31% (27,34)
Quintile 2	89% (83, 95)	79% (74, 85)	87% (81,93)	75% (68,82)	63% (57,70)	48% (42,55)	77% (74,81)	69% (65,73)	44% (41,48)	32% (28,35)
Quintile 3	89% (83, 94)	80% (75, 86)	86% (81,92)	75% (69,81)	63% (56,70)	48% (42,54)	80% (76,83)	72% (68,76)	48% (44,51)	35% (31,38)
Quintile 4	88% (82, 95)	78% (72, 84)	89% (84,94)	79% (72,85)	64% (56,71)	48% (41,55)	78% (75,82)	70% (66,75)	50% (46,54)	37% (33,41)
Quintile 5	88% (81, 94)	78% (71, 84)	87% (82,93)	77% (71,83)	66% (58,73)	51% (44,57)	79% (75,82)	71% (67,75)	49% (45,53)	36% (32,39)
**Food Security**										
Food insecure	88% (82, 95)	78% (72, 84)	87% (80,93)	74% (67,81)	63% (55,70)	47% (40,54)	80% (76,84)	73% (68,78)	43% (38,48)	31% (26,35)
Food secure	89% (83, 94)	79% (74, 85)	87% (82,93)	77% (71,82)	64% (57,70)	48% (42,54)	79% (75,82)	71% (67,74)	47% (44,50)	34% (31,37)

Results are adjusted for age, sex (children 2-8 years), misreporting, weekend/weekday and sequence of recall. CDRR in children: 1,500 mg/day; males 9-18 years: 2,300 mg/day; females 9-18 years: 2,300 mg/day; males ≥ 19 years: 2,300 mg/day; female's ≥ 19 years: 2,300 mg/day
